# LZTR1 loss-of-function variants associated with café au lait macules with or without freckling

**DOI:** 10.3389/fneur.2024.1391425

**Published:** 2024-08-27

**Authors:** Svea Horn, Teresa Neuhann, Corina Hennig, Angela Abad-Perez, Eva-Christina Prott, Lisa Cardellini, Cornelia Potratz, Jonas Leubner, Birgit Eichhorn, Martin Merkel, Angela Abicht, Angela M. Kaindl

**Affiliations:** ^1^Charité–Universitätsmedizin Berlin, Department of Pediatric Neurology, Berlin, Germany; ^2^Charité–Universitätsmedizin Berlin, Center for Chronically Sick Children, Berlin, Germany; ^3^MGZ – Medizinisch Genetisches Zentrum, München, Germany; ^4^Mitteldeutscher Praxisverbund Humangenetik, Dresden, Germany; ^5^Charité-Universitätsmedizin Berlin, Institute of Human Genetics, Berlin, Germany; ^6^Praxis für Humangenetik, Wuppertal, Germany; ^7^Charité–Universitätsmedizin Berlin, Institute for Cell Biology and Neurobiology, Berlin, Germany

**Keywords:** *LZTR1* gene, frameshift, café au lait macules, neurofibromatosis type 1, gene panel analysis, schwannomatosis

## Abstract

Pathogenic variants in the leucine zipper-like transcriptional regulator 1 gene (*LZTR1*) have been identified in schwannomatosis and Noonan syndrome. Here, we expand the phenotype spectrum of *LZTR1* variants. We identified four loss-of-function heterozygous *LZTR1* variants in five children with multiple café au lait macules and one adult with multiple café au lait macules and axillar freckling, by applying gene panel analysis in four families. The three *LZTR1* variants, namely, c.184del/p.Glu62Serfs*39, c.1927C < T/p.Gln643*, and c.857_858delinsT/p.Gly286Valfs*65, were novel, whereas the variant c.1018C > T/ p.Arg340* had been previously reported in a patient with schwannomatosis. Similar to what is known from other *LZTR1*-associated conditions, penetrance of the skin manifestations was reduced in two carriers of the familial variants. Our study expands the LZTR1 phenotype to the presence of isolated café au lait macules with or without freckling. Thus, variants in the *LZTR1* gene should be considered in patients with multiple café au lait macules.

## Introduction

Heterozygous germline variants of the leucine zipper-like transcriptional regulator 1 gene *LZTR1* (MIM*600574) have recently been reported in patients with a susceptibility to schwannomatosis 2 (MIM#615670) and also with Noonan syndrome types 2 and 10 (MIM#616564, MIM#605275).

Schwannomatosis is characterized by a predisposition to develop multiple non-intradermal schwannomas (histologically benign nerve sheath tumors) between the second and fourth decades of life (1). The penetrance is markedly reduced and is estimated at 40–50% (1). Schwannomatosis-associated *LZTR1* variants include mainly non-sense, frameshift, and splice-site variants, and loss of function is the principal disease mechanism (2). A loss of heterozygosity was found in 90% of schwannomas of *LZTR1* germline mutation carriers (2). Skin manifestations, such as café au lait macules or freckling, have not been previously reported (1).

Noonan syndrome is a group of phenotypically similar developmental disorders that are caused by pathogenic variants in multiple genes all linked to the RAS/MAPK signaling pathway. This condition is mainly characterized by developmental delay, short stature, typical facial dysmorphisms, and heart defects. In this syndrome, café au lait macules are part of the clinical spectrum. Pathogenic variants in *LZTR1* leading to Noonan syndrome can be inherited in an autosomal dominant and autosomal recessive manner. Autosomal dominant Noonan syndrome-causing pathogenic variants affect specific positions in the Kelch domain (3). Biallelic loss-of-function variants of *LZTR1* are the cause of autosomal recessive Noonan syndrome (4).

*LZTR1* encodes the leucine zipper-like transcription regulator 1, a Golgi protein that belongs to the BTB-Kelch superfamily. It has been identified as a tumor suppressor gene. Somatic *LZTR1* variants have been associated with numerous cancers such as glioblastoma and breast and liver cancer (5). LZTR1 facilitates the polyubiquitination and degradation of RAS via the ubiquitin–proteasome pathway, leading to the inhibition of the RAS/MAPK signaling (6–8). Dysregulation of the RAS/MAPK pathway is a hallmark of diseases known to have multiple café au lait macules, such as neurofibromatosis type 1 (NF1, MIM#162200), caused by *NF1* variants and Legius syndrome (MIM#611431) caused by sprout-related EVH1 domain-containing protein 1 (*SPRED1*) variants (9). Importantly, somatic and germline mutations of *LZTR1* trigger oncogenesis by dysregulated ubiquitylation-dependent lysosomal degradation, accumulation, and activation of epidermal growth factor receptor (EGFR) and AXL, two oncogenic receptor tyrosine kinases (5). LZTR1-mutant tumors exhibit vulnerability to concurrent inhibition of EGFR and AXL, thus offering a targeted therapy approach (5).

Here, we report a cohort of six affected individuals, five patients aged 3 months to 3.5 years with multiple café au lait macules and a mother of two of these patients affected with not only café au lait macules but also freckling. These otherwise healthy individuals came from four unrelated families and carried different stop and frameshift variants in *LZTR1*. These data support the inclusion of *LZTR1* in gene panels for suspected NF1 due to café au lait macules with or without freckling.

## Clinical report

**Figure 1:**
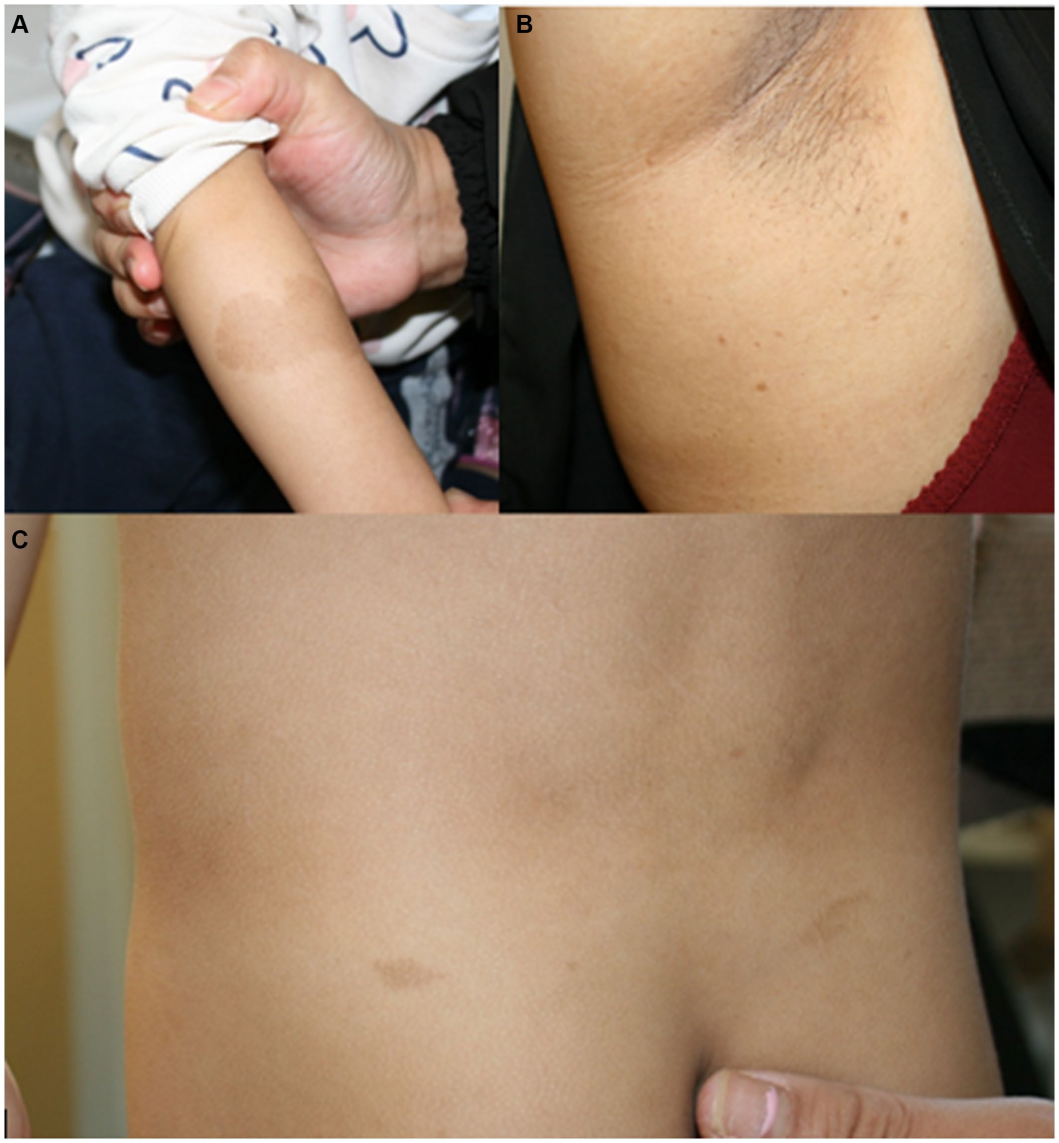
**(A)** Café au lait macule on the right arm of patient 1 at the age of 2.5 years. **(B)** Axillar freckling in the mother of patient 1. **(C)** Café au lait macules on the back of patient 4 at the age of 3.5 years.

**Figure 2:**
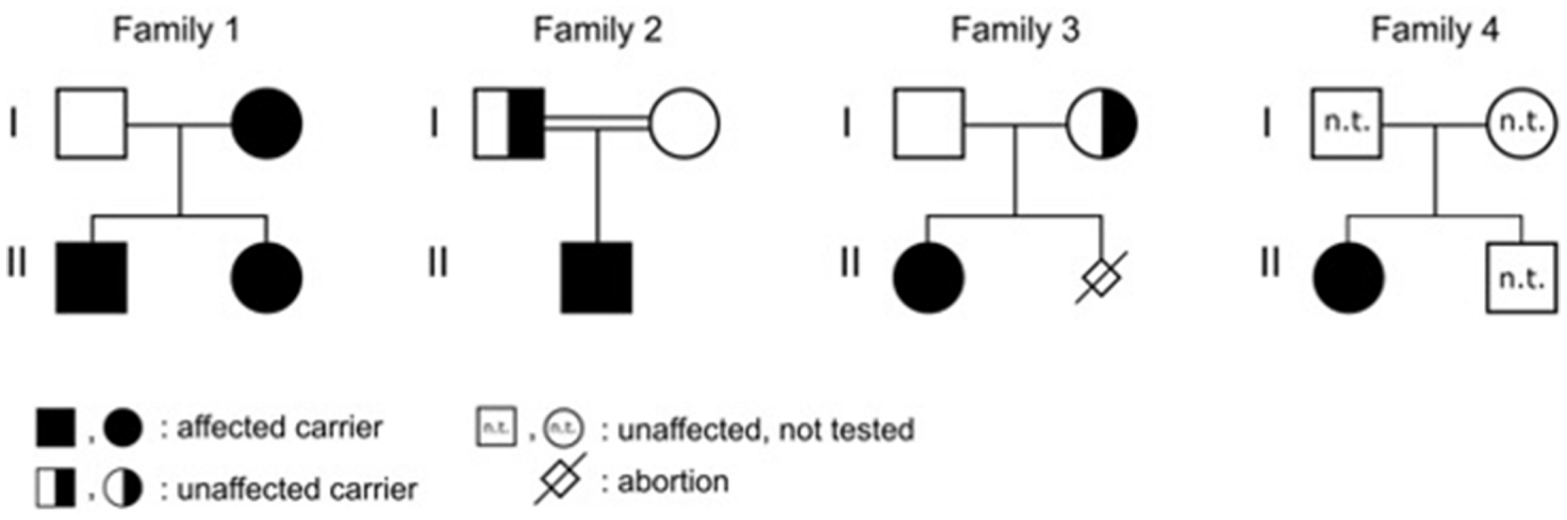
Pedigrees with marked affected individuals.

*Patient 1* was a 21-month-old daughter of non-consanguineous parents of Bangladeshi origin (Figures 1, 2). She was born preterm after an uneventful pregnancy at 35 + 5 weeks of gestation. Multiple café au lait macules were observed in the neonatal period. At the time of examination at 21 months, her height was 82.4 cm (22nd centile, −0.77 SD), weight 11.6 kg (50th centile, 0.0 SD), and occipitofrontal head circumference (OFC) was 47.8 cm (46th centile, −0.10 SD). She exhibited seven café au lait macules with a size of more than 0.5 cm and two additional smaller café au lait macules, no skinfold freckling, and no facial dysmorphisms (Figure 1, Table 1). Her psychomotor development was normal. Echocardiographic investigation at the age of 2 years excluded a congenital heart defect or cardiomyopathy. Gene panel analysis, including the genes *NF1, NF2, SPRED1, SMARCB1,* and *LZTR1*, identified a likely pathogenic variant in the *LZTR1* gene, c.1018C > T, p.Arg340* (NM_006767.4). This variant had been reported previously in one individual with schwannomatosis and is predicted to result in a stop codon and truncation of the protein.

**Table 1:** Clinical and molecular findings in the affected individuals.

Patients	Patient 1 (family 1)	Patient 2 (family 1)	Patient 3 (family 1)	Patient 4 (family 2)	Patient 5 (family 3)	Patient 6 (family 4)
Origin	Bangladesh	Bangladesh	Bangladesh	Turkey	Germany/Thailand	Turkey
Sex	Female	Female	Male	Male	Female	Female
Genotype in *LZTR1*	c.1018C > T; p.Arg340*	c.1018C > T; p.Arg340*	c.1018C > T; p.Arg340*	c.184del; p.Glu62Serfs*39	c.1927C < T; p. Gln643*	c.857_858delinsT; p.Gly286Valfs*65
ACMG classification	Likely pathogenic	Likely pathogenic	Likely pathogenic	Likely pathogenic	Likely pathogenic	Likely pathogenic
Age at evaluation	21 months	34 years	12 months	2.5 years	3 months	10 months
Clinical signs	>6 CALM	Axillar freckling, CALM	>6 CALM	>6 CALM	>6 CALM	>6 CALM

Examinations of the healthy mother (*patient 2*) (Figure 2), who was a carrier of this likely pathogenic variant, revealed bilateral axillar freckling, two café au lait macules of more than 1.6 cm diameter, and two smaller café au lait macules on her trunk and legs (Figures 1, 2). The 12-month-old brother (*patient 3*) (Figure 2) of the index patient, who also harbored this variant, showed normal psychomotor development and normal growth parameters (Figure 2). Examination at this age revealed six café au lait macules of more than 0.5 cm diameter and multiple smaller café au lait macules on his trunk and legs.

*Patient 4* was the 2.5-year-old son of consanguineous parents of Turkish origin (Figure 2). The family history was unremarkable. He was born at term without complications after an uneventful pregnancy, with normal birth weight (3,370 g, 22nd centile, −0.78 SD), birth length (51 cm, 19th centile, −0.88 SD), and OFC (36 cm, 54th centile, +0.11 SD). His psychomotor development was normal. He was able to sit at the age of 6 months and walk at 16 months. His first words were spoken at the age of 12 months.

At 2.5 years he showed normal growth parameters: height 94 cm (67th centile, +0.43 SD), weight 14.7 kg (77th centile, +0.73 SD), and OFC 49.3 cm (22th centile, −0.78 SD). Postnatally, the café au lait macules started to appear. Examination at 2.5 years of age revealed six café au lait spots with a diameter of more than 0.5 cm located on his trunk and legs. He had no skinfold freckling, cutaneous or subcutaneous neurofibromas, or dysmorphic facial signs (Figure 1, Table 1). Echocardiographic and ophthalmologic examinations at the same age did not reveal any abnormalities. At 3.5 years, repeated examination revealed seven café au lait macules of more than 0.5 cm diameter and 3 smaller café au lait macules. Because of the suspicion of NF1, a gene panel analysis including the genes *NF1, NF2, SPRED1, SMARCB1,* and *LZTR1* was performed. Therefore, a heterozygous likely pathogenic frameshift variant in the *LZTR1* gene, c.184del, p.Glu62Serfs*39, (NM_006767.4:) was detected. This frameshift variant led to a stop codon and caused truncation of the protein. The healthy father was a carrier of this likely pathogenic variant and did not show any skin manifestations. The mother did not carry this variant.

*Patient 5* was a 3-month-old girl, the first child of non-consanguineous parents of German and Thai origin, respectively (Figure 2). The first pregnancy of the mother ended in a spontaneous abortion. The further family history was unremarkable. The patient was born at term after an uneventful pregnancy with a normal OFC of 33 cm (7th centile, −1.4 SD). Postnatally, multiple café au lait macules developed. Examination at 3 months revealed normal growth parameters [length 59 cm (48th centile, −0.05 SD), weight 5.8 kg (68th centile, +0.45 SD), OFC 38.1 cm (7th centile, −1.48 SD)], 6 café au lait macules distributed over the integument, no freckling, and no dysmorphisms (Table 1). At the age of 17 months, approximately 30 café au lait macules were present, two in the size of 2 × 15 mm, and the other in an average size of 4 mm. The girl’s psychomotor development was normal. Gene panel analysis excluded pathogenic variants of the *NF1, NF2, SPRED1,* and *SMARCB1* genes and revealed the heterozygous likely pathogenic variant c.1927C < T, p. Gln643* in the *LZTR1* gene (NM_006767.4), which results in a premature stop codon. This variant has not been described in the literature or databases (ClinVar, HGMD, gnomAD). According to the ACMG classification, this variant was classified as a class 4 variant (10). The mother who did not show any skin manifestations or other clinical signs also carried this variant. The father was excluded as a carrier of this variant.

The affected individual of family 4 (*patient 6*) was a 10-month-old girl born to healthy Turkish parents with one additional healthy son (Figure 2). The further family history was unremarkable. At 38 + 2 weeks of gestation, her birth length (49 cm, 24th centile, −0.71 SD), birth weight (2,895 g, 22nd centile, −0.78 SD), and OFC (33 cm, 14th centile, −1.07 SD) were normal. After 4 months of life, multiple café au lait macules were noticed. Her psychomotor development was in the normal range with speaking first words at 11 months and free walking at 15 months. At the age of 10 months, examination showed 13 café au lait macules, strabismus, and no freckling or facial dysmorphism. At 2 years, height (85 cm, 32nd centile, −0.47 SD), weight (12.5 kg, 63rd centile, +0.33 SD), and OFC (47 cm, 14th centile, −1.06 SD) were in the normal range. Gene panel analysis including the *NF1, NF2, SPRED1,* and *SMARCB1* genes identified a likely pathogenic variant c.857_858delinsT, p.Gly286Valfs*65 in the *LZTR1* gene, which results in a premature stop codon. This variant has not been reported in the literature and has been classified as class 4. A segregation analysis could not be performed in this family.

## Methods

Based on isolated genomic DNA from peripheral blood, a next-generation sequencing analysis, using targeted enrichment for library preparation and the NovaSeq6000 System (Illumina, San Diego, CA), was performed in patients 1 and 5. The results were verified by Sanger sequencing (ABI 3730 capillary sequencer) after PCR amplification. The sequencing data were analyzed with SeqPilot (JSI medical systems). Segregation analysis was performed by Sanger sequencing in the parental samples of both families and the affected brother of patient 1.

The DNA sample of patients 4 and 6 was extracted from peripheral blood. Whole exome enrichment was performed with the Twist Human Comprehensive Exome Kit (Twist Biosciences). Massively parallel sequencing was carried out on a NovaSeq 6,000 system (Illumina, San Diego, CA) as 150-bp paired-end runs using v1.5 SBS chemistry. Exome enrichment-based SNV, INDEL, and CNV calling was conducted using varvis® 1.19.3 (Limbus Medical Technologies GmbH, Rostock). A multi-gene panel including the neurofibromatosis and schwannomatosis-associated genes (*LZTR1, NF1, NF2, SMARCB1,* and *SPRED*) was evaluated. Only SNVs and small INDELs in the coding and flanking intronic regions 106 (±50 bp) were evaluated. The analysis of the parental samples of patient 4 was performed equally by whole exome enrichment and the Twist Human Comprehensive Exome Kit (Twist Biosciences). The evaluation was focused on and targeted at the *LZTR1* variant detected in the index patient. Variants were classified according to the ACMG guidelines in all index patients (10).

## Discussion

We present the clinical and molecular data of four unrelated families with four loss-of-function *LZTR1* variants, thereby expanding the LZTR1 phenotype to the presence of isolated café au lait macules with or without freckling. Thus, *LZTR1* variants should be considered as a differential diagnosis in patients with café au lait macules. The *LZTR1* variant c.1018C > T, p.Arg340*found in this study has previously been reported in association with schwannomatosis in a single patient without further clinical signs (11). The three other variants (c.184del, c.1927C < T, and c.857_858delinsT) in this study were novel.

Six or more than six café au lait macules were observed as the only manifestations in the five patients with *LZTR1* variants in infancy and toddler age, and freckling combined with café au lait macules was present in one adult carrier (Table 1, Figures 1, 2). Due to these features, they meet one clinical criterion for neurofibromatosis type 1 and prompted us to initiate the molecular diagnosis of the *NF1* gene and related disorders. The future appearance of further clinical signs, e.g., schwannomas, cannot be ruled out because the clinical manifestations in schwannomatosis and related disorders are clearly age-dependent.

In a large Italian cohort (*n* = 250) of suspected NF1 patients investigated by targeted next-generation sequencing, the pathogenic *LZTR1* variant c.154-154delC, p.His52Ilefs*49 was identified in a 12-year-old girl presenting with only 15 café au lait macules and her father with multiple schwannomas (12). In the same study, two other affected individuals were found to carry, in addition to a pathogenic *LZTR1* variant, a second pathogenic variant in another gene (the *NF2* gene in one patient and the *PTPN11* gene in the other), resulting in a possible alternative explanation for the cause of multiple café au lait macules observed in both. In addition to the diagnosis of schwannomatosis, two café au lait macules were detected in two further adult patients with two different pathogenic variants in *LZTR1* in the same study (12).

Differential diagnoses, such as *LZTR1-related* Noonan syndrome or somatic mosaicism, for NF type 1 are not probable in the mutation carriers reported in the present study. Clinical signs of Noonan syndrome, such as short stature, developmental delay, distinctive facial features, and congenital heart defects, were not present in any of the carriers of the *LZTR1* variants. The distribution of café au lait macules extended over the entire bodies of these affected individuals and was not in a restricted region; therefore, somatic mosaicism for NF type 1, which cannot be captured by testing blood lymphocytes, is not likely.

Markedly reduced penetrance in *LZTR1-associated* schwannomatosis is well known. We also hypothesize a reduced penetrance for the clinical sign “café au lait macules” in two of the four families reported here as not all carriers of the here described *LZTR1* variants (father of patient 4 and mother of patient 5) showed clinical signs. Regarding café au lait macules, reduced penetrance was also notable within two of four reported *LZTR1-related* Noonan syndrome families (13). The published literature on Noonan syndrome patients caused by pathogenic variants of *LZTR1* has been reviewed by Farncombe et al. (13). They documented that in 4 of 48 reported families with the different types of Noonan syndrome associated with *LZTR1* variants, affected individuals had café au lait macules in addition to their other disease features.

Individuals with monoallelic germline loss-of-function variants of *LZTR1* have clearly been documented with an increased risk of developing schwannomas in adulthood. However, loss-of-function *LZTR1* variants were also documented in gnomAD, a database of the general population, which could be interpreted that carriers of loss-of-function variants have a lower risk of symptomatic schwannomas than in a typical autosomal dominant disorder (2). A comparison of the frequency of loss-of-function *LZTR1* variants between schwannomatosis patients and the general population displayed a markedly reduced risk of schwannomas compared to *NF2* and *SMARCB1* pathogenic variants (2).

A common dysregulated RAS/MAPK pathway as the consequence of mutations of different components of this pathway, such as the LZTR1 and the NF1 proteins, may result in overlapping clinical spectrums of associated disorders. One patient with LZTR1-related Noonan syndrome who showed an overlapping phenotype between Noonan syndrome and schwannomatosis was recently reported (13). Furthermore, a pathogenic germline loss-of-function variant of *LZTR1* was also described in a patient affected with not only multiple schwannomas but also neurofibromas demonstrating an overlapping clinical spectrum of conditions caused by different altered genes of the RAS/MAPK pathway as in this case schwannomatosis and neurofibromatosis (14).

Current clinical management for schwannomatosis recommends that individuals have a baseline brain and spine MRI in late childhood/early adulthood to monitor the disease (1). Future investigations of the targeted therapy approach with EGFR and AXL inhibitors in *LZTR1*-altered tumors and schwannomas are of high relevance (5).

Our data strengthen the association of loss-of-function *LZTR1* variants and multiple café au lait macules as the only manifestation in infancy and toddler age as well as freckling as an additional manifestation in adulthood. We assume a reduced penetrance for these skin manifestations, in parallel to other conditions related to *LZTR1* variants, such as schwannomatosis. Based on our findings, the inclusion of the *LZTR1* gene in panels of neurofibromatosis-related genes should be considered. An alternative approach for the future may be trio exome sequencing, capturing comprehensive genetic data.

In conclusion, our data expand the differential diagnoses in children with multiple café au lait macules and establish a link to the *LZTR1* gene. Further clinical and molecular data are necessary to confirm this association.

## Data Availability

The original contributions presented in the study are included in the article/supplementary material, further inquiries can be directed to the corresponding author.
